# Preclinical evaluation of reversible pulsed electrical field: electrophysiological and histological assessment of myocardium

**DOI:** 10.3389/fcvm.2024.1426920

**Published:** 2024-08-01

**Authors:** Zongwang Zhai, Yuchen Ling, Yanjiang Wang, Liang Shi, Xingpeng Liu

**Affiliations:** ^1^ Department of Cardiology, Beijing Chaoyang Hospital, Capital Medical University, Beijing, China; ^2^ Department of Cardiology, Peking University Shougang Hospital, Beijing, China; ^3^ Department of Research and Development, Shanghai HT Co. Ltd., Shanghai, China

**Keywords:** precise ablation, electroporation, reversible pulsed electric fields, critical isthmus sites, para-Hisian arrhythmia, sinus node modification

## Abstract

**Background:**

Pulsed field ablation, as a non-thermal ablation modality, has received increasing attention. The aim of this study is to explore whether a reversible pulsed electric field (RPEF) can temporarily inhibit electrical conduction and provide a novel method for precise ablation of arrhythmia.

**Methods:**

RPEF energy was delivered from an ablation catheter to the atrium of six dogs, followed by a series of electrogram and histology assessments.

**Results:**

RPEF ablation of ordinary myocardium resulted in an average reduction of 68.3% (range, 53.7%–83.8%) in electrogram amplitude, while 5 min later, the amplitude in eight electrograms returned to 77.9% (range, 72.4%–87.3%) of baseline. Similarly, the amplitude of the sinoatrial node electrograms reduced by an average of 73.0% (range, 60.2%–84.4%) after RPEF ablation, but recovered to 84.9% (range, 80.3%–88.5%) of baseline by 5 min. No necrotic change was detected in histopathology. Transient third-degree atrioventricular block occurred following the ablation of the maximum His potential sites with RPEF, the duration of which was voltage dependent. The histopathological results showed necrosis of the myocardium at the ablation sites but no injury to His bundle cells.

**Conclusions:**

RPEF can be applied to transiently block electrical conduction in myocardial tissues contributing to precise ablation.

## 1 Introduction

Pulsed field ablation (PFA), is a non-thermal ablation modality, which leverages a continuous microsecond-level high-voltage electric field to induce irreversible electroporation (IRE), thereby disrupting cell membrane stability and ultimately leading to cell death (1). Of note, PFA has been preclinically and clinically confirmed to have certain tissue specificity, for ablating myocardium with little effect on some adjacent non-cardiac tissues (e.g., the esophagus and phrenic nerve) (2–8). PFA has attracted great attention because of its potential safety and has been used in some clinical trials to treat atrial fibrillation (AF) (9–14).

Different from the irreversible electroporation of PFA, a low intensity pulsed electric field (PEF) can reversibly electroporate cells (1, 15, 16). Attenuated pulses can affect the transient permeability of the cell membrane without impacting dielectric breakdown of the cell membrane and cell death, which contributes to introducing various biomaterials (such as impermeable drugs and genetic materials) into cells (17–21). Deliberately applying such sublethal electric pulses to the myocardium can induce temporary changes in the electrical properties of the myocardium without causing permanent injury to the myocardium.

Accordingly, we hypothesized that reversible PEF (RPEF) can temporarily change the electrical properties of local myocardium and transiently suppress electrical conduction, then it can be changed to irreversible PEF ablation for accurate ablation—once critical isthmus sites of arrhythmia are confirmed. To verify this hypothesis, we studied the effect of RPEF on canine atrial tissue and we also probed into its impacts on sinoatrial node (SA) and His bundle.

## 2 Materials and methods

**Figure 1:**
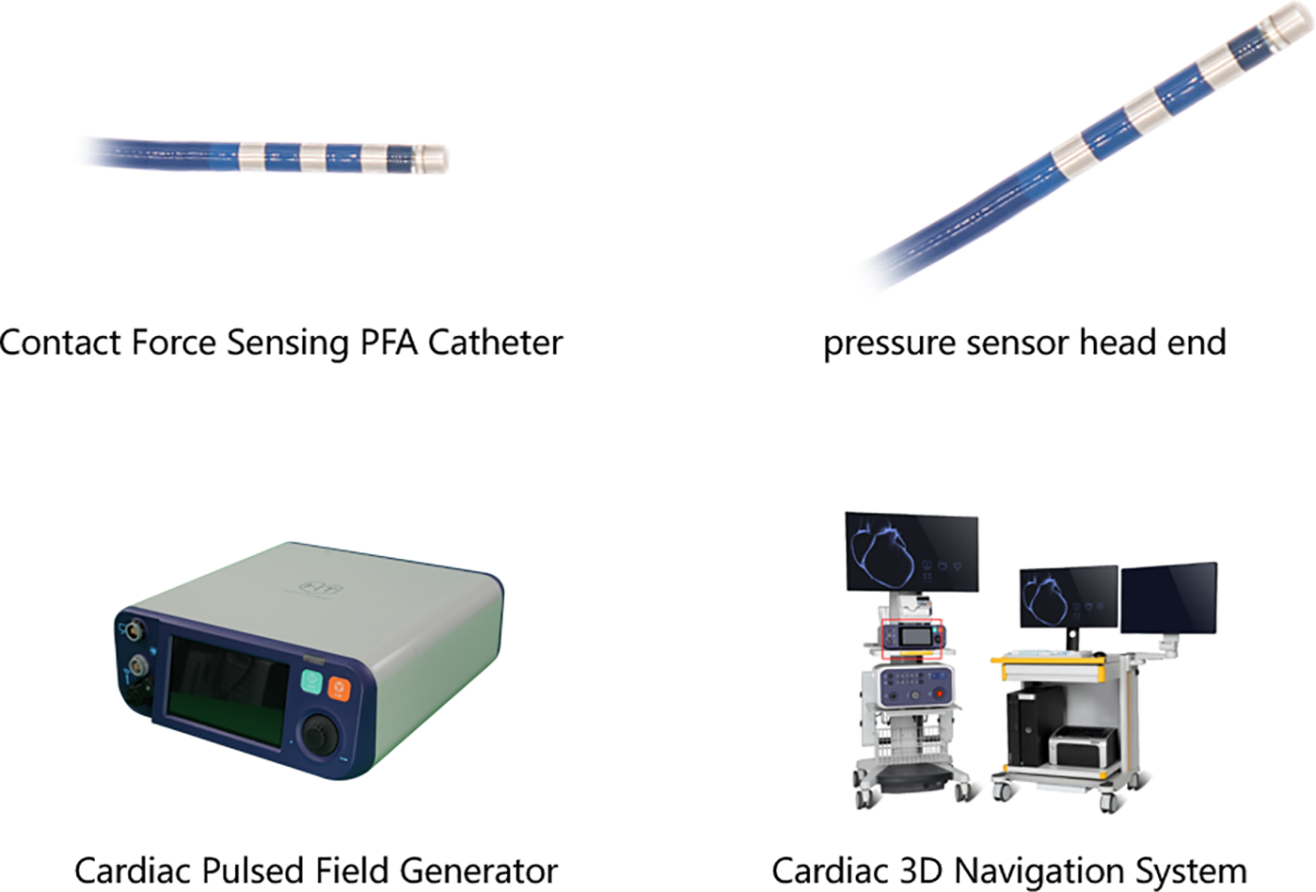
APT Cardiac Pulsed Field Ablation System.

The animal experiments were approved by the Animal Experiment and Experimental Animal Welfare Committee of Capital Medical University (Ethics number: AEEI-2023-325). The experiments involved six male Labradors (30–40 kg), and included equipment from APT Medical [Shenzhen, China (Figure 1)], encompassing the following: Cardiac Pulsed Field Generator, Contact Force Sensing PFA Catheter, Radiofrequency Ablation Generator, Radiofrequency Catheter, Cardiac 3D Navigation System, and so forth. The non-saline perfusion pressure-sensing pulse ablation catheter was of 7.5 F size, with four equally spaced electrodes at the tip, D-2 discharge, unidirectional 180° bending, and equipped with a magnetic sensor. The tip was equipped with a pressure sensor (fiber-optic sensing mode). The main application scenarios were point-by-point, linear ablation.

### 2.1 Preparation before experiment

After an overnight fast, Labradors were injected intravenously with Zoletil® 50 (Virbac, Carros, France; Tiletamine hydrochloride and Zolazepam hydrochloride at 1:1 ratio, 0.05–0.1 ml/kg) and were further injected intramuscularly with atropine (0.05 mg/kg). After intubation and the use of artificial ventilators, propofol was injected intravenously at a dose of 1 ml/min. Access to bilateral femoral veins was established.

### 2.2 Experimental procedure

#### 2.2.1 Preclinical experiment 1 (atrial myocardium)

Intracavitary echocardiography (10F SOUNDSTAR 3D Diagnostic Ultrasound Catheter, Biosense Webster, Irvine, CA, USA) was used only to guide the atrial septal puncture. Post a single atrial septal puncture, an adjustable curved sheath tube was inserted into the left atrium (LA) under the guidance of fluoroscopy. Intravenous administration of heparin maintained an active coagulation time of 300–350 s. The pressure at the tip of the pulse ablation catheter was maintained at 10–15 g, and the experiment was performed by an experienced electrophysiologist.

Under the guidance of electroanatomic mapping, the right and left atria of six Labradors were ablated by RPEF at select sites. After each ablation, the tip of the catheter was held in place in a stationary manner, and bipolar electrograms were continually recorded. The electrogram was measured continuously from the beginning of the RPEF ablation to 5 min after ablation. Electrograms before and after ablation were selected for amplitude analysis. Pacing threshold at 2 ms pulse width was evaluated at the same sites.

**Figure 2:**
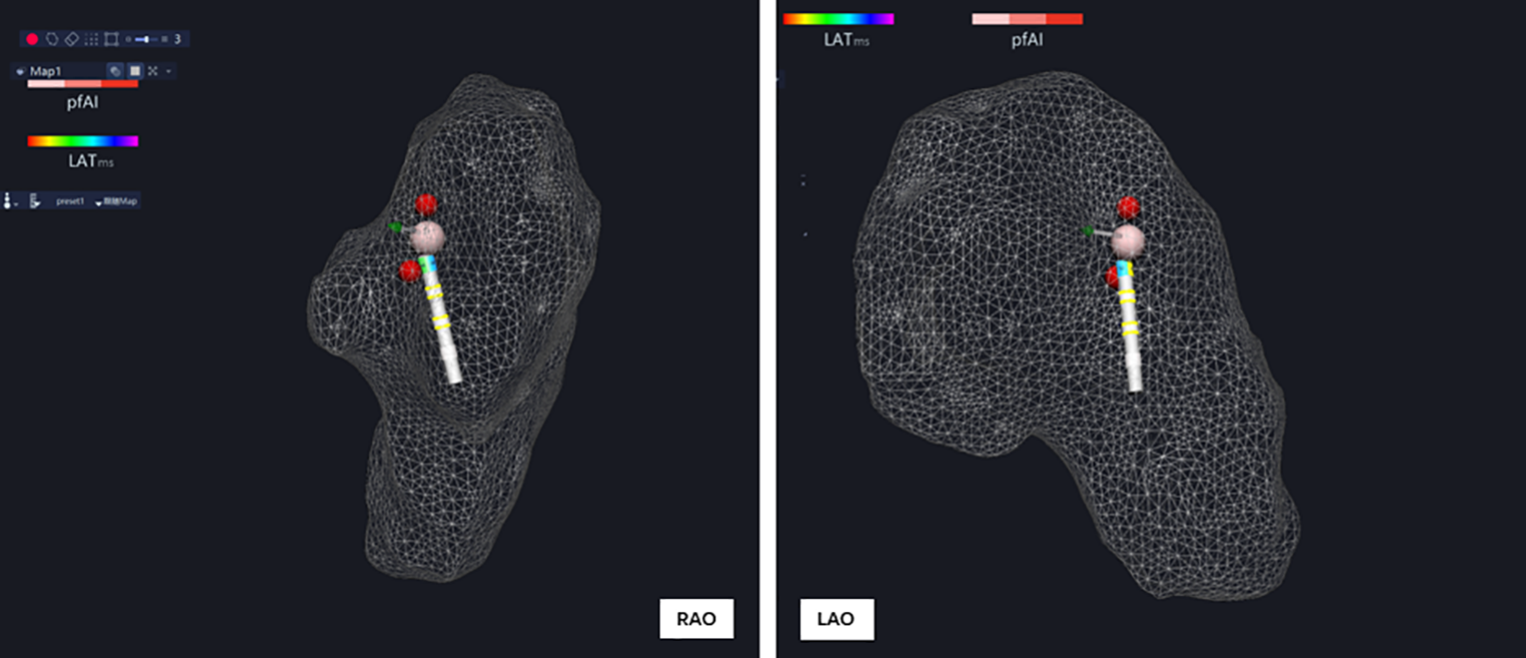
A single reversible PFA was placed between two discrete RFA lesions for identification of the RPEF lesion.

RPEF was applied between two discrete radiofrequency ablations (RFA lesions were formed 30 W, 30 s by radiofrequency catheter ablation) for identification of RPEF ablation sites at autopsy (Figure 2). The dogs were euthanized with intravenous injection of potassium chloride at a dose of 1–2 mEq/kg under basal anesthesia after 24 h. Then the hearts were extracted and stained with triphenyl tetrazolium chloride, following which all chosen sites were carefully examined and the selected tissue specimens underwent histopathological assessment. After formalin fixation, the tissues were processed and stained with hematoxylin and eosin in addition to Masson trichrome.

#### 2.2.2 Preclinical experiment 2 (His bundle)

**Figure 3:**
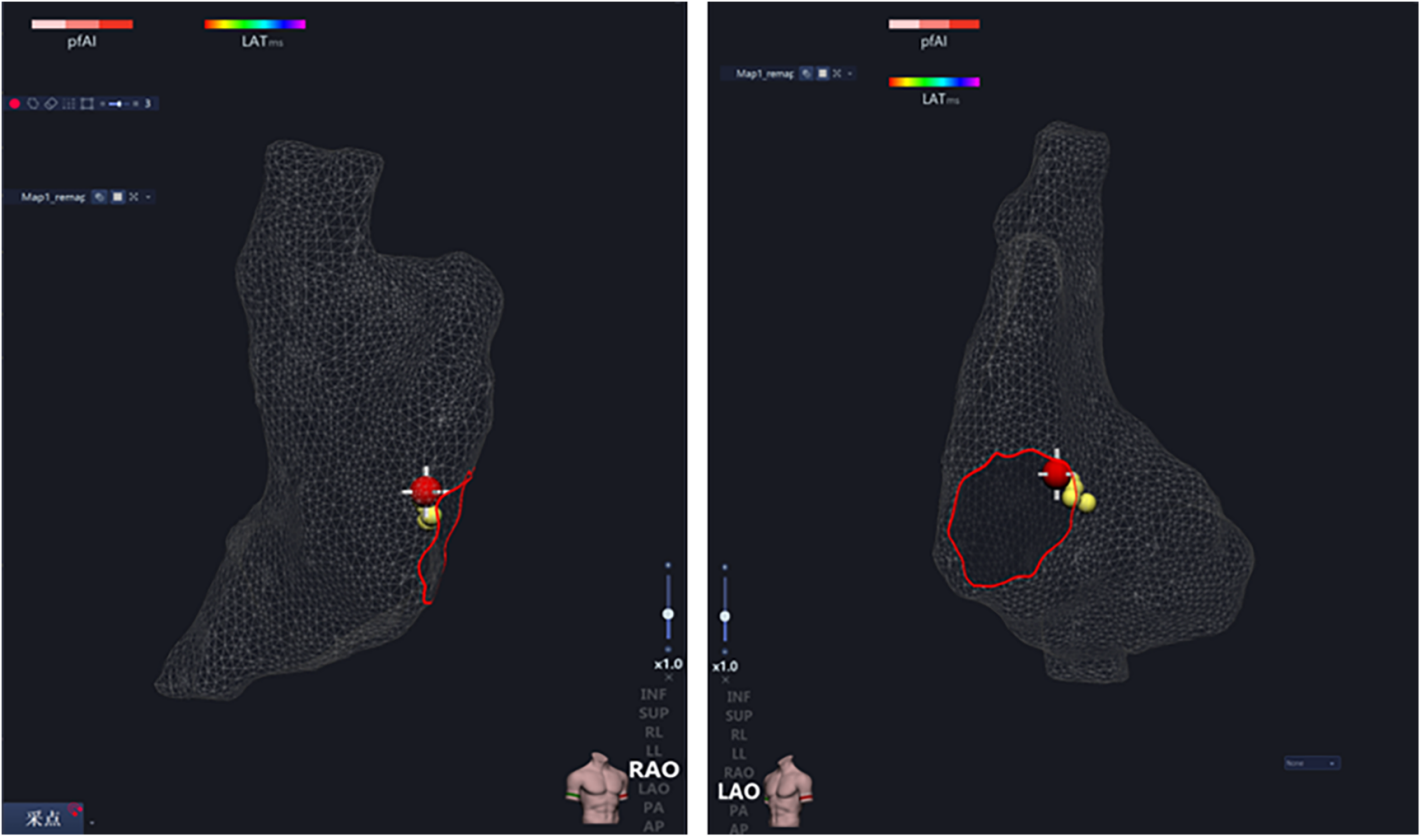
After the right atrium modeling, all His potential locations (yellow ball) were marked, and the maximum His potential location (red ball) was ablated.

Under the guidance of electroanatomic mapping, the pulsed ablation catheter was sent into the right atrium (RA) to establish the right atrium model. All His bundle potentials were marked and the maximum His potential was labeled. The maximum His potential was applied with RPEF ablation which was of micro-second pulsed width, with biphasic wave, in bipolar fashion, and one dog was ablated with one voltage to regulate the pulse electric field intensity (to avoid cumulative effect interference) (Figure 3). The recovery time of complete atrioventricular block, atrial–His (AH) interval and His–ventricular (HV) interval before ablation, and AH interval and HV interval immediately after atrioventricular block recovery were recorded.

The His bundle region was removed by anatomical localization (as described in the following) and underwent histological analysis. Discrete continuous sections were processed, and stained with hematoxylin and eosin as well as Masson trichrome.

#### 2.2.3 Preclinical experiment 3 (sinoatrial node)

**Figure 4:**
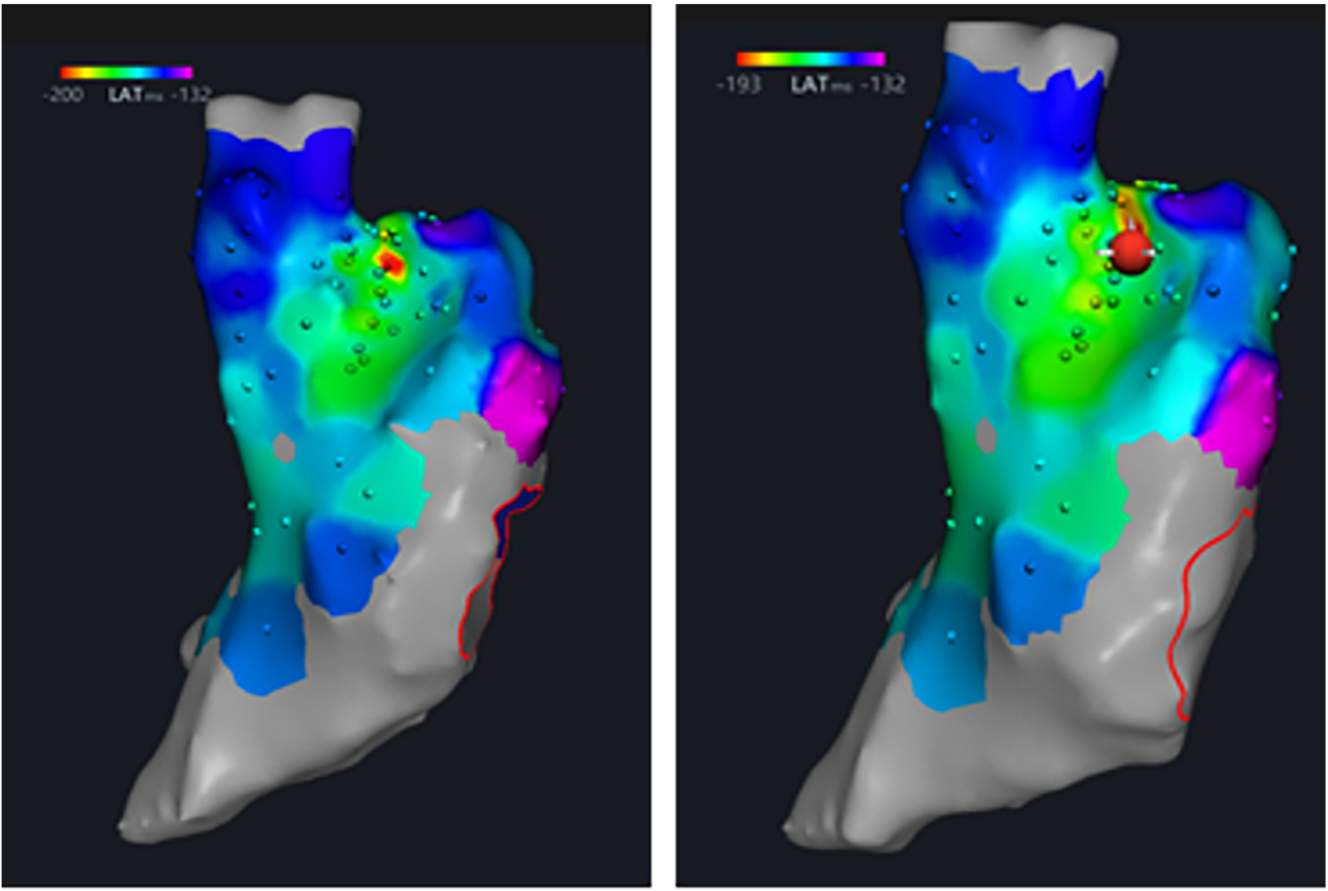
The earliest point of right atrial activation, the sinus node, was marked under the guidance of three-dimensional electrical anatomical mapping, and then was ablated with RPEF.

The earliest exciting point of right atria, the sinus node, was marked under the guidance of the three-dimensional electroanatomic mapping system, and later ablated with RPEF (Figure 4), and bipolar electrograms were continuously recorded. The function of the sinus node was assessed before and after RPEF.

After euthanasia, tissue blocks of 1 cm × 2 cm were taken from the sinus node area (as described in the following). The tissue was sliced continuously along the long axis and then stained with hematoxylin and eosin, and Masson trichrome.

### 2.3 Statistical analysis

Continuous variables are expressed as mean ± standard deviation or the median of the quartile interval, and categorical variables are described by counts and percentages. Two-group comparison was performed using the paired t-test. A value of *P* < 0.05 was considered statistically significant. SPSS 26.0 (IBM, New York, USA) software was used for statistical analysis.

## 3 Results

### 3.1 Preclinical experiment 1 (atrial myocardium)

**Figure 5:**
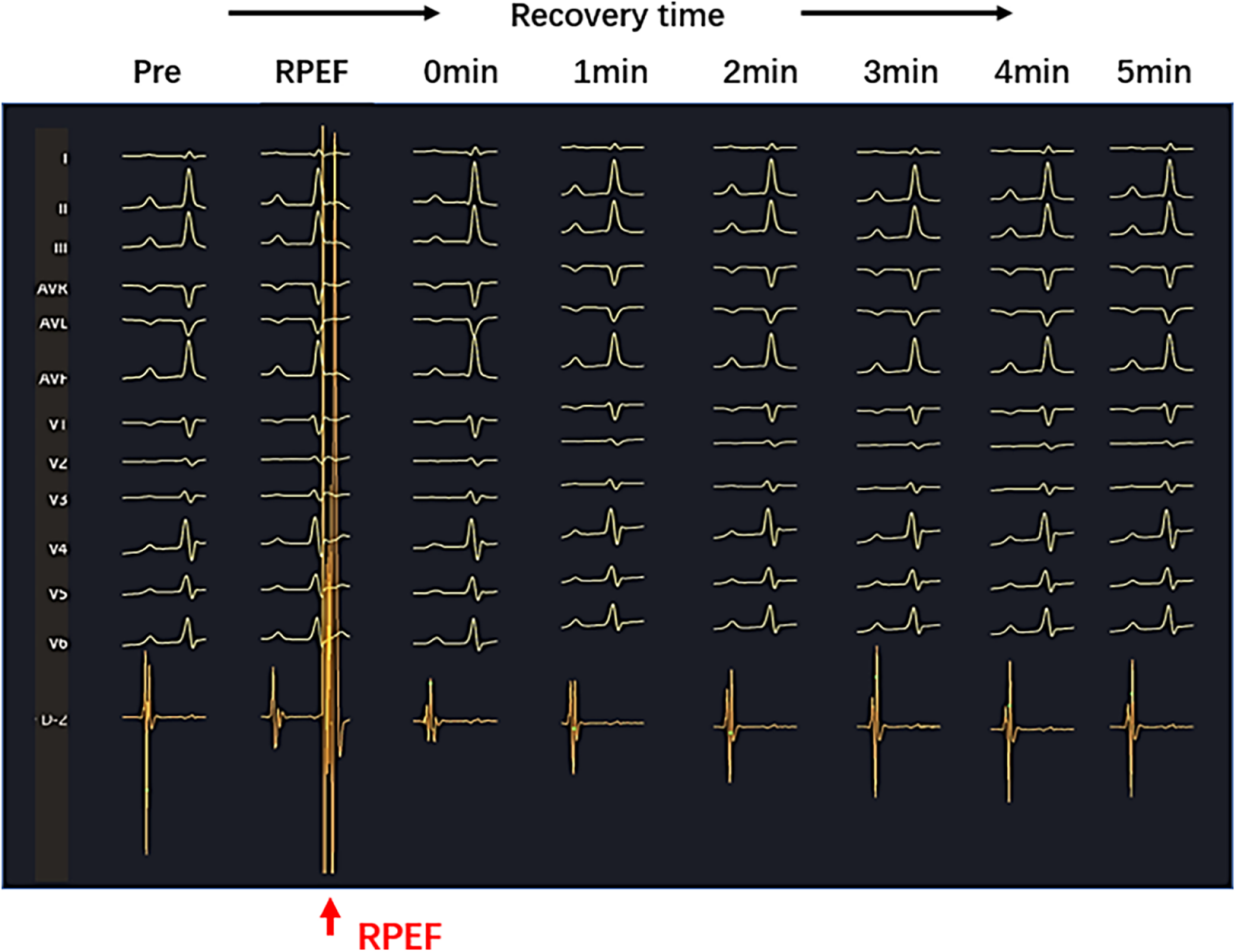
Sharply reduced electrogram (red arrow) was observed on the PEF pressure catheter after RPEF ablation. The amplitude of the electrogram decreased immediately and gradually recovered over time.

**Figure 6:**
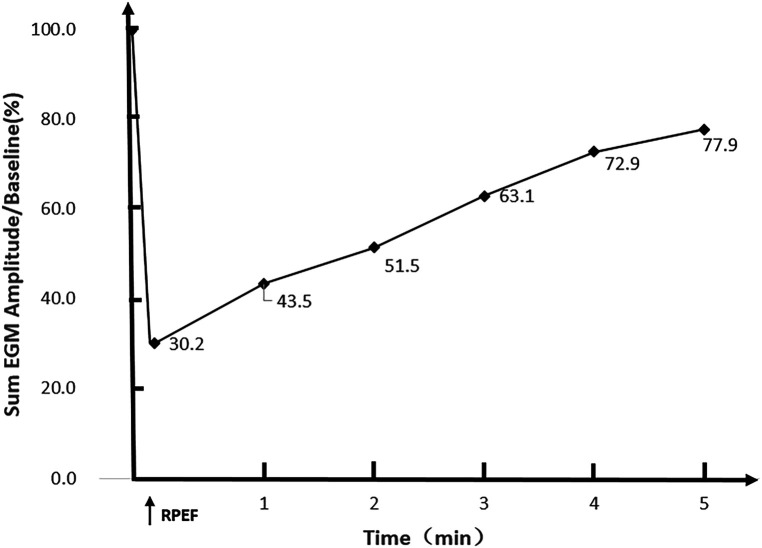
The total electrogram amplitude decrease was observed immediately after RPEF in the atrial myocardium of Labradors (*n* = 4 RPEF ablations). A sharp drop was determined in the amplitude of the electrogram followed by a gradual recovery (the horizontal axis represents time in minutes; the vertical axis shows the percentage of overall ECG amplitude recovery).

In the six dogs, eight RPEF ablation sites were detected in the posterior wall of LA (*n* = 4), the free wall of RA (*n* = 2), the RA septum (*n* = 1), and the right atrial appendage (RAA) root (*n* = 1). The baseline of electrogram amplitude was 8.1 ± 2.6 mV (range, 6.4–10.7 mV), and after ablation with RPEF, the electrogram amplitude decreased to 2.4 ± 1.5 mV (range, 1.2–3.9 mV), with a mean reduction of 68.3% (range, 53.7%–83.8%). As shown in Figure 5, the amplitude gradually recovered to 77.9% of baseline (range, 72.4%–87.3%) 5 min after RPEF ablation on eight electrograms. In Figure 6, we analyzed the electrogram amplitude per minute within 5 min after ablation at four RPEF sites and found that the total electrogram amplitude gradually recovered after a sharp decline. The electrical anatomical mapping did not reveal any bipolar low voltage (<0.1 mV) region at the RPEF ablation site. The pacing threshold was 1.1 ± 0.2 mA (range, 1.0–1.2 mA) before ablation, 3.7 ± 2.1 mA (range, 2.0–5.8 mA) immediately after ablation, and returned to 2.3 ± 1.3 mA (range, 1.3–3.0 mA) 5 min after ablation.

**Figure 7:**
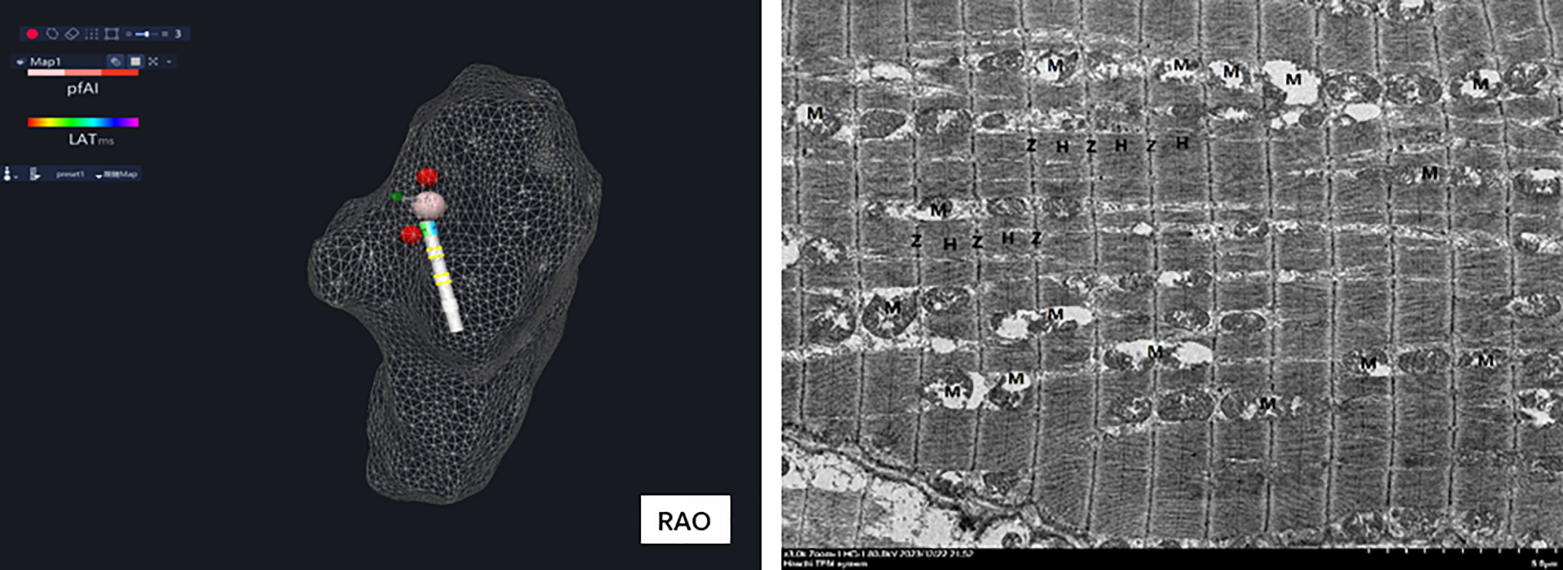
Histopathological results showed normal mild to moderate degenerative changes between two RFA lesions.

The pathological examination of the tissue between the two radiofrequency lesions (Figure 7) displayed mild to moderate degenerative changes between the two radiofrequency ablation lesions.

### 3.2 Preclinical experiment 2 (His bundle)

In the six canines, RPEFs at different voltage intensities ablated the maximum His potential locations. The baselines of AH interval and HV interval were 55.7 ± 3.9 ms (range, 52.3–59.3 ms) and 35.0 ± 1.4 ms (range, 33.8–36.3 ms), respectively. After RPEF ablation and the immediate recovery of third-degree atrioventricular blocks, AH interval was 65.7 ± 5.6 ms (range, 59.0–70.3 ms) and HV interval was 36.0 ± 2.8 ms (range, 33.8–38.0 ms). Statistics showed that the AH interval was prolonged before and after the atrioventricular blocks (*P* < 0.05), while the HV interval remained unchanged (*P* > 0.05). All six dogs presented transient third-degree atrioventricular blocks after ablation, which subsequently recovered. Under the condition that other parameters were constant, the greater the ablation voltage of RPEF was, the longer the recovery time it resulted in, reflecting the voltage-dependent characteristic.

**Figure 8:**
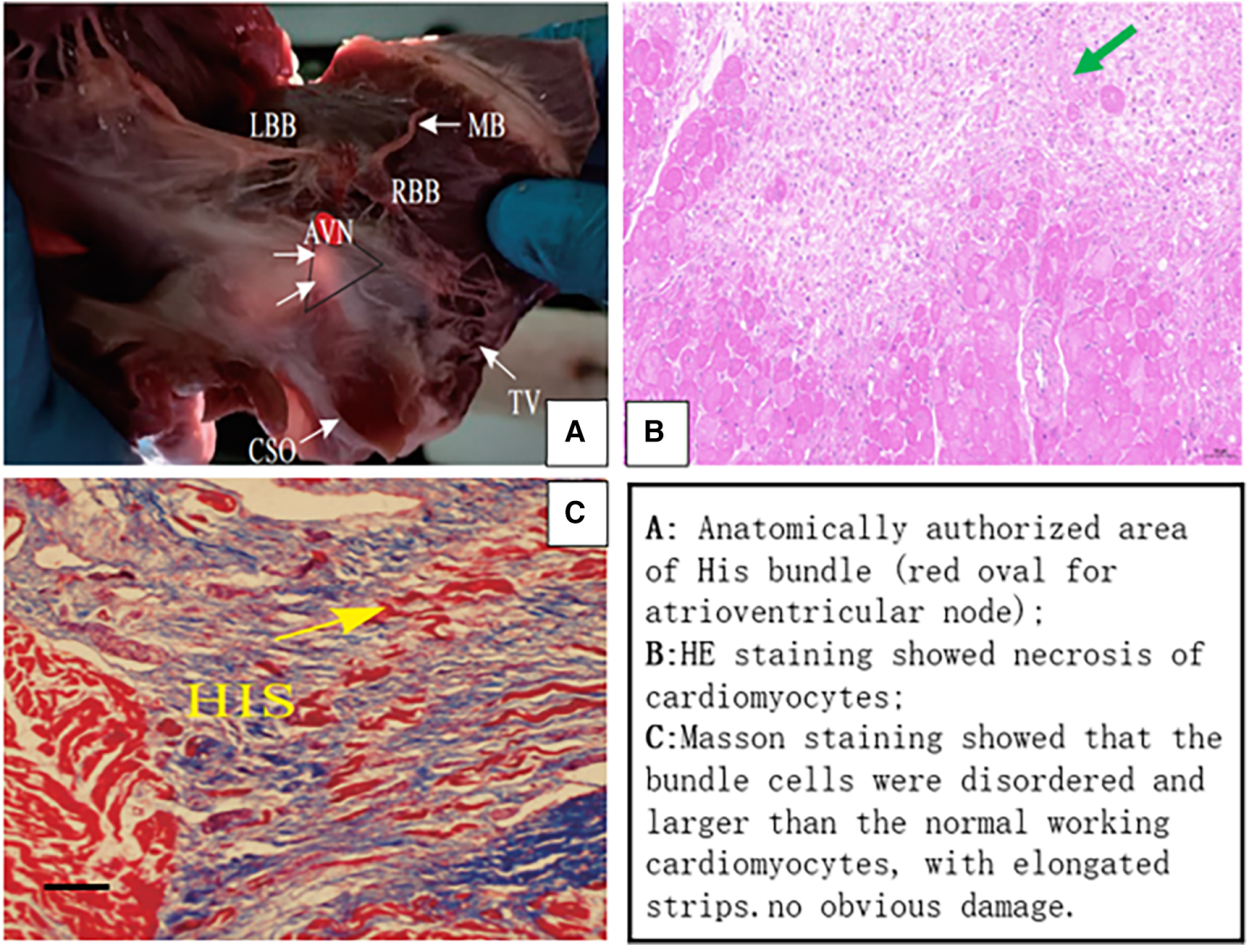
(**A**) Gross anatomical location of the His bundle and histopathology. HE staining showed local myocardial necrosis (**B**), and Masson staining exhibited normal cell structure of His bundle (**C**). AVN (red oval area), atrioventricular node; LBB, left bundle branch; RBB, right bundle branch; CSO, coronary sinus ostium; TV, tricuspid valve; MB, moderator band; HIS, His bundle.

The dogs were euthanized after interventional operation, and the position of the superior vena cava (SVC) was determined. The right atrium was incised along the superior vena cava. There is a triangular area called Koch triangle along the inner margin of the ostium of the coronary sinus (CSO), the margin of the tricuspid valve (TV), and the Todaro tendon, and the atrioventricular node area was approximately 1 cm upward along the ostium of the coronary sinus. Above the Koch triangle, the thinnest and transparent region was visible, through which the His bundle passed. The His bundle was cut out by means of local anatomy (Figures 3, 8A). Histopathological results revealed necrosis of ordinary cardiomyocytes at the ablation site, and His bundle cells were generally normal without obvious injuries.

### 3.3 Preclinical experiment 3 (sinoatrial node)

**Figure 9:**
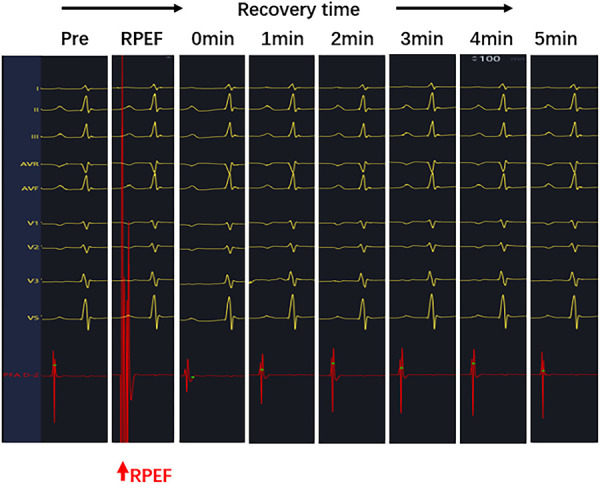
After RPEF ablation of the SA node, the amplitude of pulsed ablation catheter electrogram decreased immediately and recovered gradually over time.

The electrogram amplitude at baseline was 5.8 ± 1.0 mV (range, 4.8–6.5 mV), and RPEF caused a decrement in the electrogram amplitude to 1.5 ± 0.7 mV (range, 1.0–2.0 mV), with a mean reduction of 73.0% (range, 60.2%–84.4%), which gradually recovered to 4.9 ± 0.7 mV (range, 4.2–5.4 mV) and returned to 84.9% (range, 80.3%–88.5%) within 5 min (Figure 9). Sinus node recovery time (SNRT) before ablation was 1,410.5 ± 28.6 ms (range, 1,388.3–1,445.5 ms) and became 1,377.8 ± 31.6 ms (range, 1,349.3–1,400.0 ms) after ablation for 5 min (*P* > 0.05). The corrected sinus node recovery time (CSNRT) before ablation was 635.7 ± 20.2 ms (range, 618.3–654.0 ms) and became 633.2 ± 16.7 ms (range, 623.8–644.0 ms) after ablation for 5 min (*P* > 0.05).

**Figure 10:**
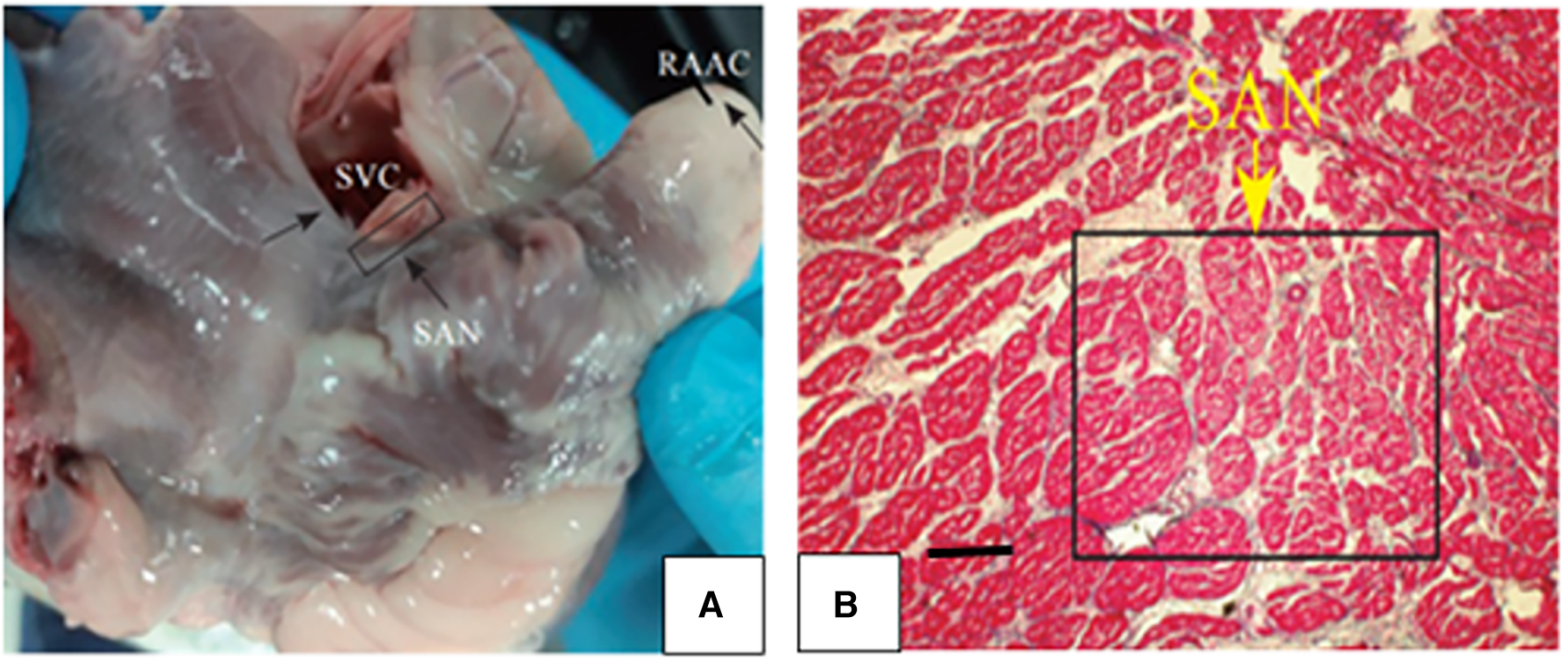
(**A**) The junction of the roots of SVC and the RAA was the sinoatrial node. (**B**) Histopathological results showed that the sinusoidal node cells were smaller and denser than the surrounding ordinary cardiomyocytes, presenting an irregular oval shape, disorderly arrangement, clumpy distribution and no clear boundary with ordinary cardiomyocytes.

The positions of the SVC and RAA were determined. The junction of the root of SVC and RAA was the sinus node (Figures 4, 10A), where tissues of 1 cm × 2 cm were obtained. Then the tissues were sliced continuously along the long axis. The histopathological results unveiled that the structure of SA node cells was generally normal with no obvious injury.

## 4 Discussion

### 4.1 Reversible effect of pulsed electric field

When RPEF energy was delivered to canine atrial tissue, the biphasic electrogram amplitude dramatically diminished by approximately 70%, followed by a gradual recovery to about 80% of baseline within 5 min. The results of the pathological analysis showed that the ablation site was nearly normal myocardium without lesion. Before arrhythmia ablation, partially reversible pulses can be delivered to potential targets to help identify critical isthmus sites. It manifests that RPEF can act on potentially important sites to terminate tachycardia, and thus contributes to identifying critical isthmus sites prior to ablation. Nowadays invasive electrophysiological mapping is widely performed in tachycardia patients. Mapping techniques such as excitation and entrainment are often used to map arrhythmia loops and identify critical isthmus sites for catheter ablation. However, these techniques fail to provide the precise location of ablation. In general, the putative target can only be determined based on whether the ablation lesion terminates the tachycardia. A lack of localization specificity results in the radiofrequency energy to possibly ablate cardiac tissue unrelated to arrhythmia.

### 4.2 Histiocytic selectivity of PEF

It has been documented that the electroporation thresholds of PEF differ greatly among different cell types (Table 1) (22–28), and PEF is particularly suitable for cardiac ablation since cardiomyocytes have the lowest electroporation threshold of all tissues (29). The difference in sensitivity between cardiomyocytes and other non-target tissues may reduce the risk of collateral damage to the esophageal and phrenic nerves, while other thermal ablation methods, such as radiofrequency and cryoballoon ablation, may damage the esophageal and phrenic nerves. The exact mechanism that makes cardiomyocytes more sensitive to lower PEFs is not fully understood, but may be related to cell size, orientation, membrane properties, and sensitivity to non-specific cation entry (30). Acute kidney injury due to hemoglobinuria after pulsed electric field ablation has also been studied. More than 70 applications seem to have better sensitivity and specificity to predict hemolysis (31–34). However, a maximum of four applications per dog were performed and the PEF intensity was reversible, considering the low likelihood of causing hemoglobinuria and acute kidney injury associated with hemolysis.

**Table 1:** IRE thresholds for various cell types.

Tissue	IRE threshold (V/cm)	References
Nerve	3,800	Li et al. (22)
VSMC	1,750	Maor et al. (23)
RBC	1,600	Bao et al. (24)
Liver	700	Sano et al. (25)
Kidney	600	Neal et al. (26)
Pancreas	500	Arena et al. (27)
Myocardium	400	Kaminska et al. (28)

VSMC, vascular smooth muscle cell; RBC, red blood cell.

Experimental studies regarding the impact of PEF on animals have indicated that there are marked differences in the effects of PEF on different cardiomyocyte types under the same pulse parameters. A monophasic pulse wave with a voltage of 1,000–1, 500 V was used to ablate the bilateral ventricular septum, which was the initial distribution of His, left and right bundle branches. It was found that 63% of experimental animals developed transient third-degree atrioventricular blocks, with a dose-dependent duration and severity, 38% had right bundle branch block, and histopathological results confirmed that Purkinje's nucleus and ultrastructure remained intact (35). In addition, a study on the isolated Langendorff model of canine heart has confirmed that the ablation mode was set with monophasic wave, 750∼2,500 V voltage, 90 μs pulse width, and 10 increments in voltage to release PEF, which could achieve irreversible damage to Purkinje's potential. The voltage required for the left bundle branch potential block was 2,000 V, whereas 2,500 V voltage only caused a few seconds of His bundle potential block (36). In our experiment, the maximum His potential was ablated with PEFs at different voltages. Transient third-degree atrioventricular blocks occurred in all dogs with biphasic wave, voltage of 600–1,800 V, the duration of which was significantly dose dependent. The structure of His bundle cells remained intact, while local ordinary cardiocytes were necrotic.

### 4.3 PEF ablation of para-His arrhythmia

When PEF was applied to the maximum potential of His bundle within a range of voltages, acute His injury and transient complete atrioventricular blocks appeared, and the higher the voltage, the longer the recovery time. The results of the pathological analysis showed that ordinary cardiomyocytes were necrotic at the ablation site, while the cells of His bundle were almost normal. Before ablation of para-His tachyarrhythmia, delivering PEF with safe voltages to the critical sites of para-His tachyarrhythmia can help determine the risk of a potential atrioventricular block, suggesting that PEF at safe voltages applied to important anatomic sites can facilitate the identification of potential risk of atrioventricular blocks prior to ablation. When patients with para-His arrhythmia receive invasive electrophysiological mapping, they are often treated simultaneously with titration ablation therapy. When electrophysiological examination confirms that there is obvious bundle potential or the ablation therapy is ineffective at the right heart target site, a femoral artery puncture is required to retrograde into adjacent sites, such as the non-coronary cusp and right coronary cusp for targets mapping, then the ablation catheter is sent to the corresponding target site for ablation. Some patients need combined ablation for the femoral artery access of the left heart and femoral vein access of the right heart. However, these ablation therapies can only be performed anatomically as far away from the His bundle as possible. In general, the risk of ablation can only be observed by the presence of atrioventricular blocks. The absence of advance prediction on ablation risk results from the energy that may injure His bundle and thus cause the conduction block.

### 4.4 Sinus node modification

Our study verified that PEF ablation with a certain voltage could cause reversible injury to the electrical conduction of the SA node, and the histopathological results showed that the SA node cell structure was normal without obvious injury. Accordingly, when the sinus node is ablated close to the target heart rate in sinus node modification, RPEF ablation can be chosen to prevent excessive ablation and avoid severe SA node dysfunction. SVC isolation can be achieved with a segmental ablation approach at the level of the SVC–RA junction in paroxysmal AF patients with non-pulmonary vein (PV) triggers arising from SVC (37). The sinoatrial node was ablated only once per dog and the pulse electric field intensity was reversible, so the possibility of SVC isolation was slim. In addition, studies have shown that PEF can be performed without damaging the phrenic nerve or causing vascular stenosis (38–41). Consequently, phrenic nerve injury and superior vena cava syndrome can be avoided when PEF is chosen as the energy modality in the sinus node modification.

### 4.5 Limitations

Nevertheless, there is still room for improvement in this study. This study only evaluated the effect of RPEF on myocardial tissue in healthy dogs, and only obtained preliminary exploratory findings. The energy intensity that can be applied to preclinical and clinical arrhythmias still needs further exploration. In addition, the sample size of this study is small, and further studies are needed to verify the completely reversible energy intensity. The limited observation periods (<5 min) after RPEF precluded an understanding of the time duration needed for complete recovery of electrogram amplitude, pacing thresholds, and AH interval. Furthermore, because PFA can be altered with even minor changes to delivery parameters, these results cannot be regarded to be applicable to other PFA systems. Other preclinical and clinical studies reported data regarding PFA systems using different parameter compositions and catheter electrode designs are needed.

## 5 Conclusion

This study demonstrated that PEF could be transmitted in a reversible manner *in vivo*, and its transient effects on cell excitability contributed to elucidating the physiological mechanism of the tachycardia circuit. We also studied acute SA node injury and acute His injury, providing new strategies for SA node modification and para-His arrhythmia ablation. Collectively, RPEF provides a novel route to accurately ablate arrhythmias.

## Data Availability

The original contributions presented in the study are included in the article/Supplementary Material, further inquiries can be directed to the corresponding author.
